# Additive Manufacturing of Co_3_Fe Nano-Probes for Magnetic Force Microscopy

**DOI:** 10.3390/nano13071217

**Published:** 2023-03-29

**Authors:** Robert Winkler, Michele Brugger-Hatzl, Lukas Matthias Seewald, David Kuhness, Sven Barth, Thomas Mairhofer, Gerald Kothleitner, Harald Plank

**Affiliations:** 1 Christian Doppler Laboratory—DEFINE, Graz University of Technology, 8010 Graz, Austria; 2 Graz Centre for Electron Microscopy, 8010 Graz, Austria; 3 Institute of Physics, Goethe University, 60438 Frankfurt, Germany; 4 Institute for Inorganic and Analytical Chemistry, Goethe University Frankfurt, Max-von-Laue-Str. 7, 60438 Frankfurt, Germany; 5 Institute of Electron Microscopy, Graz University of Technology, 8010 Graz, Austria

**Keywords:** additive direct-write manufacturing, 3D nano printing, focused electron beam induced deposition, nanomagnetic, magnetic force microscopy

## Abstract

Magnetic force microscopy (MFM) is a powerful extension of atomic force microscopy (AFM), which mostly uses nano-probes with functional coatings for studying magnetic surface features. Although well established, additional layers inherently increase apex radii, which reduce lateral resolution and also contain the risk of delamination, rendering such nano-probes doubtful or even useless. To overcome these limitations, we now introduce the additive direct-write fabrication of magnetic nano-cones via focused electron beam-induced deposition (FEBID) using an HCo_3_Fe(CO)_12_ precursor. The study first identifies a proper 3D design, confines the most relevant process parameters by means of primary electron energy and beam currents, and evaluates post-growth procedures as well. That way, highly crystalline nano-tips with minimal surface contamination and apex radii in the sub-15 nm regime are fabricated and benchmarked against commercial products. The results not only reveal a very high performance during MFM operation but in particular demonstrate virtually loss-free behavior after almost 8 h of continuous operation, thanks to the all-metal character. Even after more than 12 months of storage in ambient conditions, no performance loss is observed, which underlines the high overall performance of the here-introduced FEBID-based Co_3_Fe MFM nano-probes.

## 1. Introduction

Magnetic Force Microscopy (MFM) is an advanced Atomic Force Microscopy (AFM) operation mode that can be used to map local magnetic fields at sample surfaces, e.g., for studying magnetic thin film materials [1,2], vortices [3], skyrmions [4], or other nanoscale peculiarities [1]. The technology relies on a magnetic AFM tip, which interacts with the magnetic stray fields from the sample by using a two-pass technique: the first pass acquires the height profile in tapping mode, after which the tip is lifted to the so-called lift-height. During the second pass, the tip follows the previously gathered height profile at constant lift height. By that distance, the short-range Van der Waals forces subside, while magnetic tip-sample forces start to dominate. Varying magnetic interactions with the tip then alter the effective resonance frequencies, which in turn lead to a phase shift, forming the actual MFM image. The decisive component for MFM measurements, however, is the quality of the magnetic tip, which must meet a number of requirements. Traditionally, MFM probes are based on non-magnetic standard AFM tips that are subsequently coated with magnetic material. While high-quality magnetic coatings are available and mostly used, this added layer naturally increases the apex radius to more than 35 nm [5], which consequently reduces lateral resolution capabilities [6]. Additionally, such coatings contain the risk of delamination, which can lead to varying magnetic sensitivities or unwanted lateral offsets between morphology and MFM maps, further reducing reliability. The consequent solution would be coating-free, fully magnetic, and nano-sharp tips, which formed the motivation for this study.

Within the small pool of relevant fabrication technologies, focused electron beam-induced deposition (FEBID) is a potential candidate due to the strong progress made in recent years [7]. In principle, this additive manufacturing technique utilizes a focused electron beam for the local dissociation of functional precursor molecules. The latter are typically organometallic complexes, which are injected in gaseous states in the vacuum chamber of a scanning electron microscope (SEM) by a fine capillary needle. The electrons interact with the surface-adsorbed molecules, which leads to fragmentation into volatile parts (pumped away by the vacuum system) and non-volatile fragments that form a solid deposit on the surface. Depending on the precursor type used, the deposit can have different functionalities, including electrically insulating, semiconducting, conductive, or magnetic, as relevant for this study. Besides, the main process parameters, primary beam energy, and beam current, have a significant influence on the chemical composition, microstructure, and shape of the deposits. Beyond additive direct-writing of high-resolution quasi-1D/-2D patterns [8,9,10] or bulky 3D structures, a stationary beam exposure results in vertical nanowires with typical diameters below 50 nm and apex radii down to the sub-10 nm regime [11]. With appropriate control of the lateral beam movement, 3D nanoprinting of even complex geometries becomes possible in a predictable and reliable way [12,13]. By that, (3D) FEBID has successfully been applied for mask repair [14], sensing applications [15,16], plasmonics [17], nano-magnetics [18,19], and scanning probe applications [7]. Concerning the latter, FEBID is well suited due to several advantages: (1) structures can be precisely grown on almost any surface morphology, including pre-finished cantilevers or existing tips [20,21]; (2) flexible 3D designs [12] can be realized to either adapt nano-tips to individual requirements, as demonstrated by Jaafar et al. for Co- and Fe-base nanoprobes [6], or even advanced nano-probe concepts can be realized, as demonstrated for magnetic resonance force microscopy [22], scanning thermal microscopy [23], or electrical modes [24]; and (3) FEBID allows for sharp apexes in the sub-10 nm regime [11,23] on a regular basis to enable high-resolution AFM imaging [23,24]. Concerning MFM tips in particular, FEBID has previously been used for pillar-shaped tips for MFM probes, mostly performed with single material precursors (Fe, Co) [6,25,26,27,28].

In this work, the focus lies on the development of specially designed MFM tips from a heterometallic precursor, HCo_3_Fe(CO)_12_ [29], which reveals high metal contents (>80 at%) right after deposition at standard conditions [19,29] and excellent magnetic properties [19,30,31]. The study starts with the confinement of primary electron beam parameters, complemented by 3D design aspects to fulfill the strong demands for stable AFM/MFM operation. Optimized 3D nano-probes are then characterized via transmission electron microscopy (TEM) for structural and chemical insight. Next, MFM measurements are conducted and benchmarked with commercially available products, which demonstrates the excellent performance of FEBID-based nano-probes. Finally, we focus on quality aspects concerning wear and long-time aging, where no performance decrease is found after one year. The study is rounded out with material tuning procedures, as often performed in the past, including electron beam curing, thermal annealing, and e-beam-assisted purification in water vapor. As it will be shown, the novel MFM probes developed here reveal strong advantages over most existing MFM tips and pave the way for further high-performance AFM tips, as predicted in previous literature [7].

## 2. Results and Discussion

### 2.1. Design

**Figure 1:**
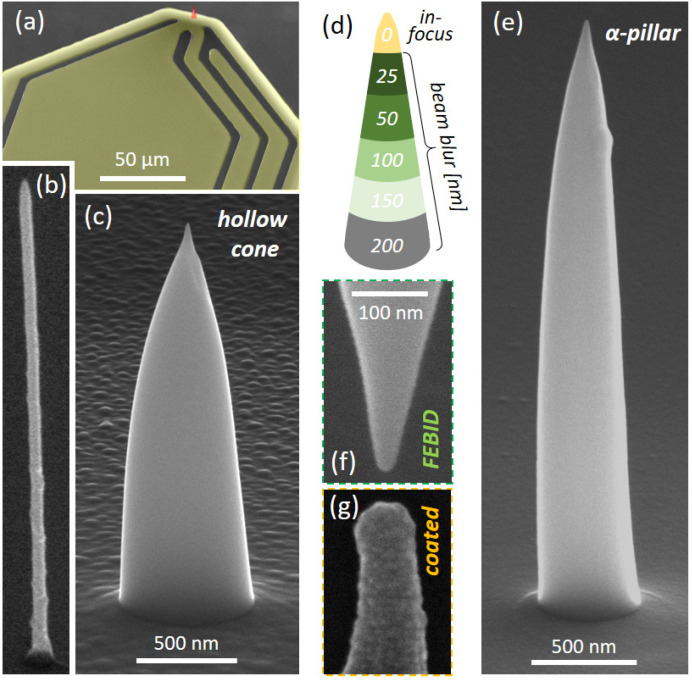
Design aspects of FEBID-based Co_3_Fe MFM tips. (**a**) tip-less SS-CL with a flexible electrode system (yellow) to adapt to different AFM probe concepts (Nice CL, GETec Microscopy) [39], revealing a nano-tip at the very end (red). While (**b**) shows a single pillar, produced by a static e-beam (5 keV, 5.2 pA, same scale bar as in (**e**)), (**c**) gives a 3D-nano printed Co_3_Fe hollow cone (20 keV, 53 pA, 1250 s) following a previous CAFM concept [24]. (**d**) shows the α-pillar patterning scheme, where the initial BB of 200 nm changes stepwise to in-focus conditions. (**e**) shows the result for such a sequence, with the same TETs as for the hollow cone in (**c**) (ETs were 500-250-200-150-100-50 s). (**f**) shows a SEM image of an α-pillar apex (r~10 nm) in direct comparison to a commercial MFM tip (MESP, Bruker), where the radius reduction is evident (**g**) (same scale bar as in (**f**)).

The demands for ideal MFM tips are listed in the introduction and require a novel design of the probe, as discussed in the following. In this special case, we aim on the fabrication on flat, tip-less self-sensing cantilever (SS-CL) platforms [32,33,34] (Figure 1a), which are equipped with specially designed electrodes (shaded yellow) to provide a general basis for a variety of AFM nano-probe concepts, ranging from magnetic over electric [24] towards thermal sensors [23]. The missing tip, however, leads to the requirement of a total tip height of at least 3 µm to ensure that the nano-probe is the lowest point of the SS-CL, which is inclined by about 11° after mounting. The simplest design is a vertical pillar, deposited under static e-beam exposure, as demonstrated in the past [6,7,35]. Although such designs can be deposited from HCo_3_Fe(CO)_12_ (denoted as Co_3_Fe) precursor, as shown in Figure 1b, the targeted length leads to mechanical challenges during AFM operation, including buckling [35] and occasional breakage/detachment, as observed during the pre-experiments in this study.

Recently, a FEBID-based, Pt hollow-cone concept was introduced for conductive-AFM (CAFM) tips on the same SS-CL platforms [24], which provides a few advantages: (1) sufficient contact area to the SS-CL to prevent detachment, (2) enhanced mechanical rigidity for AFM operation, (3) sharp tip apexes of 10 nm or less for high-resolution imaging, and (4) the single material character to eliminate delamination aspects, as relevant here as well. Figure 1c shows the feasibility of such hollow cones for the Co_3_Fe precursor, although vertical growth rates were much lower than for Pt-based materials (exact factors strongly depend on the exact deposition parameters [24]). This leads to longer processing times on the one hand, but it also entails morphological challenges due to shadowing effects [24,36]. As lateral forces during MFM are drastically lower compared to contact mode operation in CAFM, the large interface area is not absolutely needed, which would require complex patterning procedures for controlled growth. Together with the long processing times, an alternative is considered, which was introduced by Kuhness et al. [11]. The process is based on a stationary, but strongly defocused e-beam, which changes stepwise towards in-focus conditions at the very apex, as conceptually shown in Figure 1d. The advantages of that approach are: (1) enhanced volume growth rates (VGR), as areal current densities are reduced for defocused beams, which shift the working regime towards more favorable conditions [11] and, by extension, higher VGRs [37,38]; and (2) simpler patterning, as only a sequence of stationary points with varying beam blurs (BB) and respective exposure times (ET) has to be defined. Figure 1e shows an example of such a solid cone design, further denoted as α-pillar, which was grown with the same total exposure time (TET) as the hollow cone (1c). This concept allows for sharp apex radii down to 10 nm, as shown in Figure 1f in direct comparison to a commercial MFM tip (MESP, Bruker) [5], where the additional coating leads to duller apexes (1 g). Although MFM measurements were also successful with hollow cones, the remainder of the study will proceed with the α-pillar design due to reduced process times, improved morphologies, and stable AFM/MFM performance.

### 2.2. Morphology

**Figure 2:**
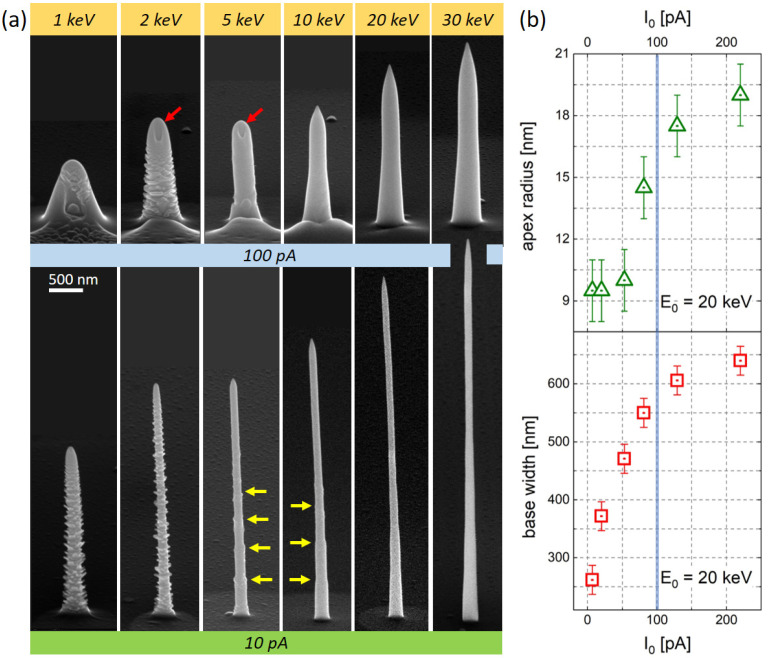
(**a**) Co_3_Fe α-pillar shapes for varying primary electron energies (increasing from left to right), fabricated at high (100 pA, upper row) and low beam currents (10 pA, lower row). The same patterning sequence was used for all fabricated α-pillars with a total exposure time of 750 s, as described in the main text. While yellow arrows indicate beam-blur steps in the morphology, red arrows indicate the indent formation. (**b**) shows the current dependent evolution of apex radii (top triangles) and base widths (bottom squares) for α-pillars produced at 20 keV, which were further used for MFM measurements.

After determining the ideal α-pillar design, the impact of process parameters on overall morphologies was evaluated, which, for 3D-FEBID architectures, is a complex interplay of a multitude of effects [40,41]. Figure 2a gives a representative selection of Co_3_Fe α-pillars, deposited with the same design (BB=150−100−50−25−0 nm; ET=150−120−90−60−330 s; TET=750 s), while the primary electron energies $E_{0}$ are varied (from left to right) for two different beam currents, $I_{0}$ of 100 pA (upper row) and 10 pA (lower row). While higher $E_{0}$ always leads to taller structures, higher $I_{0}$ results in shorter but clearly wider cones (upper row). Nominally, BB starts with 150 nm at the bottom, which is close to the found values for low currents (lower row). During further growth, vertical growth modes dominate over lateral widening, indicating a nicely balanced working regime [42]. While steps due to BB-reduction can be seen for 5 keV and 10 keV (yellow arrows), they smear out for higher energies, which can be explained by the increasing spatial distribution of forward scattered electrons (FSE). For decreasing $E_{0}$, the dissociation probability increases, which reduces local coverages and, by extension, VGRs, as evident for both currents in Figure 2a (from right to left). For higher $I_{0}$ (upper row), base diameters are getting much wider and are found clearly beyond 150 nm, as defined by the widest BBs, even when taking broader e-beams into account as well. In agreement with the arguments before, higher currents lead to stronger local depletion, which reduces vertical growth, while FSE-driven lateral growth becomes an essential part. This interpretation is further substantiated by the formation of indents (red arrows in Figure 2a) for 5 keV and below, as theoretically predicted for such situations [42]. The FSE-driven lateral widening also prevents the clear identification of BB-steps, leading to smooth surfaces for 10 keV and above. Figure 2b summarizes the current dependent evolution of α-pillar details, produced at 20 keV and further used for MFM measurements presented below. As evident, the apex radii (triangles) are around 10 nm for the lowest currents and increase to about 15 nm for 100 pA (blue vertical line), which is still much smaller compared to commercial products. Base widths start around 260 nm for the lowest currents and then increase to about 600 nm for 100 pA, which clearly lies above the 150 nm beam blur specified for the first bottom element.

Considering α-pillars for AFM operation, wider shaft regions are advantageous in terms of mechanical stability, while long and narrow pillars are prone to buckling [35] and bending under force load. Consequently, we exclude single-pillar designs [6] (Figure 1b), while low-current α-pillars (lower row in Figure 2a), though narrow, are evaluated via AFM/MFM. Primary electron energies below 5 keV are also excluded due to indent formation [42], which inherently prevents high-resolution imaging. Concerning the apex radii, 10 nm can be achieved, although 15 nm is a more relevant value considering the constraints above, which still is clearly below alternative products.

### 2.3. Functionality

**Figure 3:**
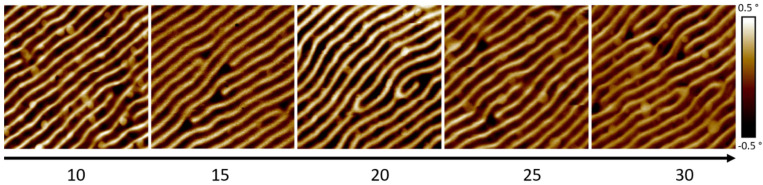
$2\times 2\;{µm}^{2}$ wide MFM phase maps of the CoPt multilayer sample, imaged with α-pillars fabricated at different $E_{0}$, while patterning design and $I_{0}$ were kept identical and similar, respectively, as specified in the main text.

Besides the shape criteria, discussed before, the material properties are decisive when applied for MFM. The degree of precursor dissociation, which impacts the chemistry as well, depends on the process parameters [43,44]. Therefore, the optimum parameters for ideal shapes are not necessarily appropriate concerning the magnetic properties of α-pillars. For the here used precursor, an almost pure Co_3_Fe core (bcc phase) with a metal-oxide sheath is assumed for optimized conditions at 20 keV [19,30]. Fortunately, that electron energy coincides with the excellent shape performance for α-pillars as shown above (see Figure 2a). The most relevant assessment, however, is provided by real MFM experiments, for which an α-pillar series was fabricated at varying $E_{0}$ and $I_{0}$. Two types of samples were used for evaluation: (1) a hard disc drive and, in particular, a CoPt multilayer system [45,46], which, for the available layer thickness, should reveal a lateral magnetic modulation in the range of 150 nm [2]. Figure 3 shows a series of MFM phase images on the CoPt sample, acquired with α-pillars and fabricated with the same pattern (BB=150−100−50−25−0 nm; ET=150−120−90−60−330 s), while $E_{0}$ was varied from 10 keV to 30 keV at similar currents around 100 pA. As evident, all nano-probes reveal fingerprint-like MFM maps with lateral modulation widths in the expected range [2]. Please note that detected MFM signals depend on the scan parameters in the second pass, foremost the lift-height, which is the tip-sample distance. To provide the highest comparability, the lift-height was first reduced until the van-der-Walls regime became dominant (a sudden phase jump), after which the distance was again increased until an artifact-free image was obtained. As Figure 3 shows MFM maps with the same phase range of ±0.5°, it becomes evident that the strongest signals are obtained at 20 keV. Together with the compositional aspects mentioned before, $E_{0}$ was fixed to 20 keV for all further experiments.

**Figure 4:**
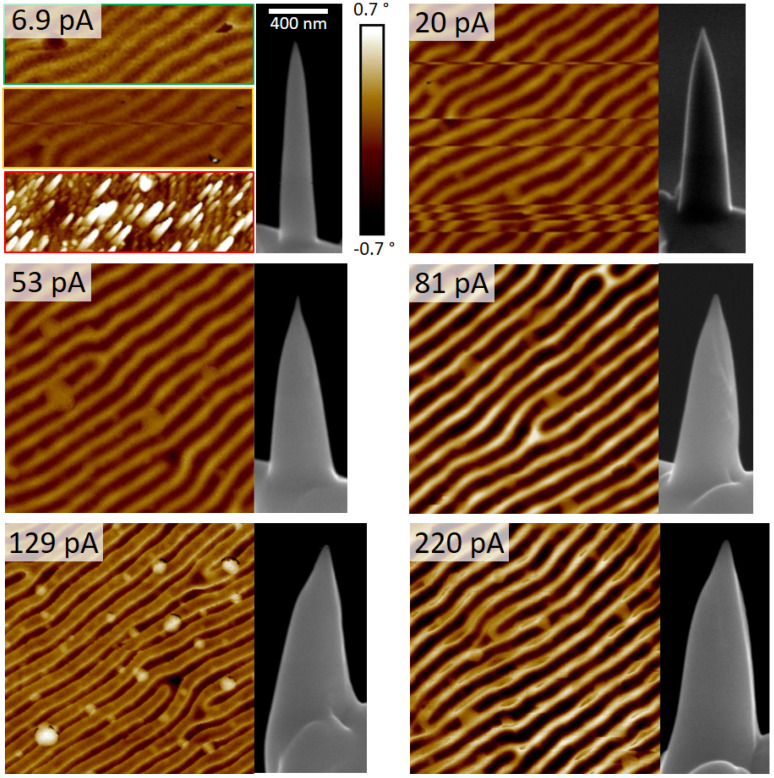
$2\times 2\;{µm}^{2}$ wide MFM phase maps of the CoPt multilayer sample, imaged with α-pillars of similar heights, fabricated at 20 keV, while $I_{0}$ was varied from 6.9−220 pA. For lowest currents, the MFM performance was very unstable, as shown by three representative images on the top left. The right SEM images show the respective Co_3_Fe probes; the scale bar of 6.9 pA applies to all.

Next, beam currents were varied from 6.9 pA to 220 pA, while ETs have been adapted to provide similar heights for all α-pillars. Figure 4 shows $2\times 2\;{µm}^{2}$ MFM phase-maps from the CoPt sample together with α-pillar SEM images. For the lowest $I_{0}$ of 6.9 pA, MFM-maps were extremely unstable, as shown by three representative images. While overall stability increased for 20 pA α-pillars, permanent contrast inversions were found, which might indicate reversals of the tip magnetization. For $I_{0}$ of 53 pA and above, no such reversals were found, and overall imaging became very stable and reliable. α-pillars fabricated at 81 pA generally revealed strong magnetic phase shifts (all images are represented in the same phase range of 1.5°), while noise decreased, providing smooth MFM-maps.

For higher currents of 129 pA, MFM maps become very sharp and change the fingerprint appearance from a sinusoidal to a more trapezoidal cross-sectional shape, which is closer to the predicted magnetic domain formation in such materials [47]. A detail worth mentioning concerns the slightly brighter edge features (129 pA in Figure 4), which might indicate that local tip-sample interaction can be very strong due to the sharp apex, entailing unwanted orientation effects at domain borders. This effect occurs even stronger for α-pillars produced at a higher current of 220 pA, which might indicate an upper limit, not least due to the fact that the apex gets larger as well (Figure 2b). By that, we confine the ideal parameter range at 20 keV to 80−120 pA, where the α-pillar design provides stable, strong, and low-noise MFM-phase signals, while apex radii are found around 15 nm, a prerequisite for high-resolution imaging.

### 2.4. Structure and Chemistry

**Figure 5:**
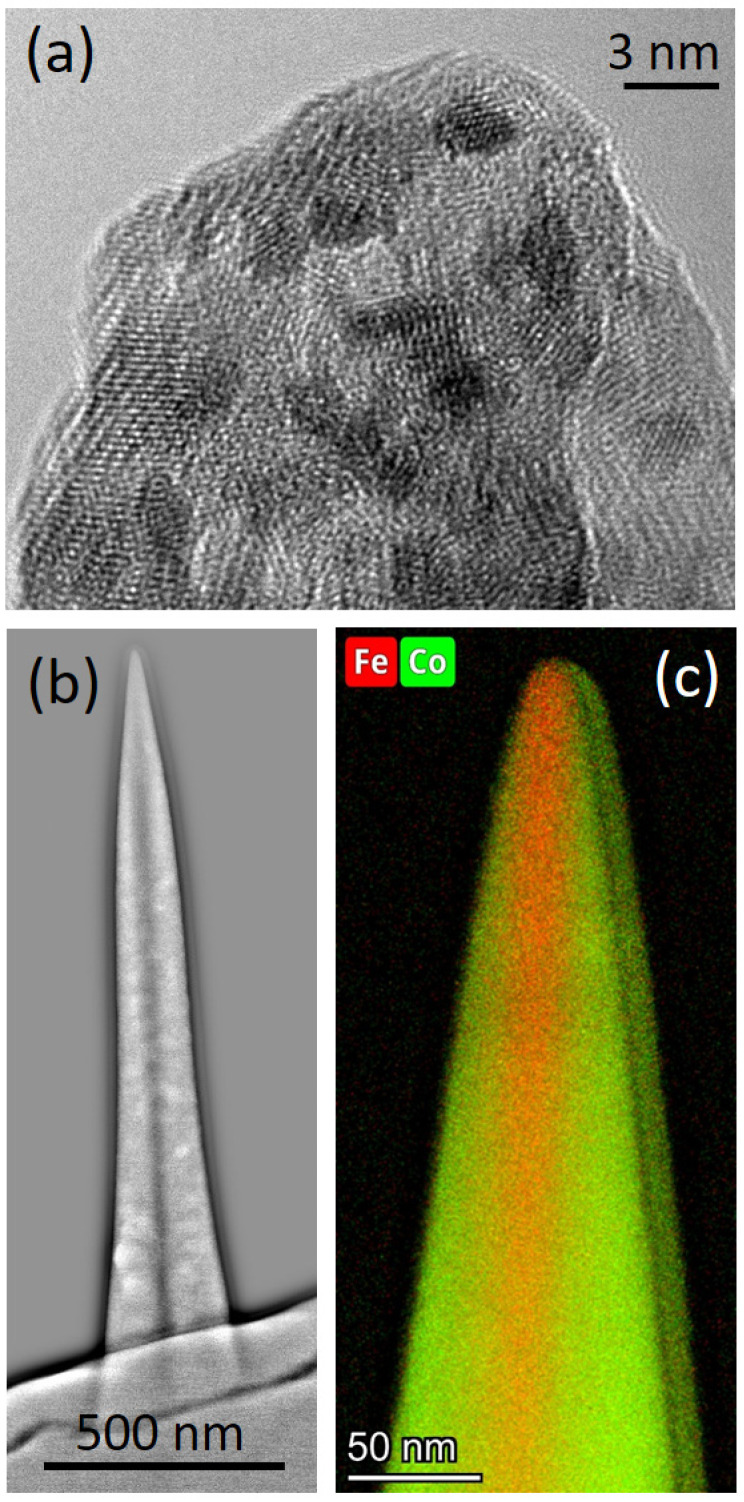
TEM characterization of Co_3_Fe pillars. (**a**) high-resolution TEM image of the tip region confirms the high degree of crystallinity, the contamination-free character, and the sharp apex. (**b**) high-pass filtered TEM-HAADF image of a 20 keV/81 pA Co_3_Fe α-pillar, which reveals a dark core in the center. (**c**) Scanning-TEM EDX map of Fe and Co distribution at the tip region, which reveals Fe-richer core, while surrounding areas are closer to the expected Co_3_Fe composition.

For a more detailed structural and chemical analysis, transmission electron microscopy (TEM) was conducted for 20 keV α-pillars fabricated at selected beam currents. As the α-pillar design inherently contains a wide basis (see the bottom panel in Figure 2b), only the upper parts allow for reliable analytical TEM results, while the lower parts only provide qualitative results. Figure 5a gives a high-resolution TEM image, in which three aspects become evident: (1) the very high crystallinity of the as-deposited material, (2) the absence of significant contamination, and (3) the sharp apex. Figure 5b shows a high-pass filtered high-angular-annual-darkfield (HAADF) image of the whole α-pillar, which revealed a dark region in the central area. Interestingly, that feature was found for all studied energies (5–30 keV) and currents (20−220 pA). This indicates either a lower average atomic number Z associated with a change in composition or a lower crystallinity/thickness. Figure 5c shows a TEM-based X-ray spectroscopy (EDX) map of the tip region, which reveals higher Fe contents in central regions while outer parts contain Co and Fe contents close to the expected Co_3_Fe composition [19,29,48].

Although a closer look at this peculiarity needs to be done in the future, we currently propose that the high areal current density at the very center of the e-beam leads to a different composition, where local beam heating can play a role as well [49,50]. In outer regions, where FSE contributions dominate and explain the pillar broadening, decomposition is closer to previous studies [19,29,48], which confirmed a Co_3_Fe composition by the application of lower currents than applied here. For completeness, we want to comment on the right-hand side of Figure 5c, which stems from the proximal co-deposition of adjacent α-pillars [51].

### 2.5. Post-Growth Procedures

As discussed before, α-pillar properties can be tuned by fabrication parameters $E_{0}$, $I_{0}$, and advanced patterning. Although results are already very promising for as-deposited materials, different post-growth procedures were tested, as they have been proven useful for other FEBID materials in the past.

First, thermal annealing was applied as it is a simple post-processing route, which partially led to significant improvements [52,53,54]. For that, 20 keV α-pillars, fabricated at currents from 6.9 pA to 220 pA, were subjected to a 3-h treatment at 250 °C and 500 °C annealing step, with SEM/AFM/MFM characterization before and after. While all nano-probes revealed a strongly reduced AFM/MFM performance after 250 °C, they become even useless for MFM after the 500 °C annealing step. SEM revealed that all pillars underwent massive broadening in all regions. Together with the fact that such a treatment is very challenging for the here-used SS-CL platforms, this type of post-processing was entirely excluded. Next, purification via post-growth e-beam exposure in H_2_O environments was considered, as it is known to effectively remove remaining carbon contents when working with platinum (MeCpPt^IV^Me_3_) [55], gold (Me_2_Au(acac), Me_2_Au(tfa)) [17,56], and to a certain extent with ruthenium (EtCp)_2_Ru) [57]. Although Co_3_Fe precursor provides more than 80 at.% metal content and for 3D structures close to pure metal after deposition [19,29,48], this procedure was applied for completeness. Here, previously reported protocols for Pt-based CAFM nano-probes [24] were used, which, however, reduced the overall performance during AFM (resolution) and MFM (signal strength), as expected. Together with the additional processing times, that kind of post-processing can be excluded. Finally, another set of α-pillars was subjected to electron beam curing (EBC) [58], which means a post-growth e-beam exposure without a precursor gas present. EBC is known to (1) proceed precursor fragmentation [58,59], which (2) leads to grain growth [60,61], while (3) cross-linking the carbonaceous matrix [15], which (4) improves the overall mechanical properties [15] and apex wear resistance [23], as essential benefits for AFM [7]. As before, the Co_3_Fe nanoprobes were pre-characterized and then subjected to EBC at 30 keV and 2400 pA for 300 s.

**Figure 6:**
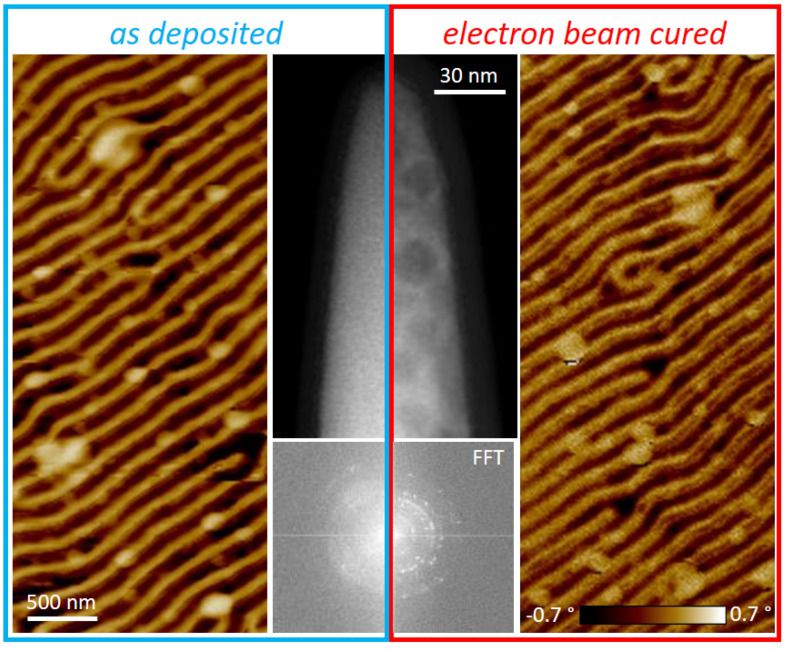
Impact of electron beam curing on Co_3_Fe α-pillars. Results (MFM phase, HAADF image, and Fast Fourier Transformation) of as-deposited tips are framed in blue, and the red box presents the results after curing. Scale and color bars apply for each image type.

Figure 6 summarizes the implications, which start with TEM-based analyses in the center. As evident by the HAADF comparison, EBC led to a significant structural variation where large darker spots appear. Fast Fourier Transformation analyses of sufficiently thin tip regions are shown below, which clearly reflect the larger grains by the change from rings (left) to more isolated spots (right). Such coalescence has been observed in the past for EBC-treated MeCpPt^IV^Me_3_ precursor [61]. Aside from structural and morphological changes, EBC led to the formation of a carbon surface layer, which stems from hydrocarbon residues in the chamber, as no precursor gas was present during EBC. Concerning MFM performance, no variation in the magnetic signal strength was observed. The details, however, have slightly changed, although no significant improvement is found. Consequently, EBC can be done but does not provide significant advantages at that point, making such a treatment not worth considering. By doing so, we have applied previously introduced post-growth procedures, although none of them provided any significant improvement. Hence, Co_3_Fe materials are well suited for AFM/MFM nano-probes right after deposition, even saving processing time during production.

### 2.6. Performance

**Figure 7:**
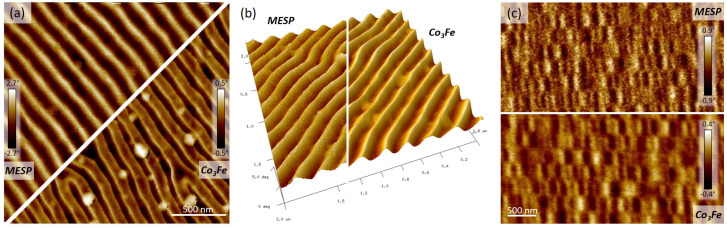
MFM-map comparison between well-established commercial MFM tips (MESP, Bruker) and FEBID Co_3_Fe α-pillars for the CoPt sample (**a**,**b**) and a magnetic hard disc drive (**c**), revealing sharper features and reduced noise. The latter is also evident in (**b**) by 3D representations of MFM maps on a CoPt sample, which are vertically adapted to achieve a similar 3D effect.

After deriving a design and identifying parameters for sensitive and stable MFM operation (see Figure 4), FEBID-based α-pillars were compared to well-established commercial nanoprobes. The latter achieve their MFM suitability through high-quality magnetic coatings, which consequently increase their apex radii, as shown in Figure 1g. In contrast, Co_3_Fe α-pillars are intrinsically composed of magnetic material, therefore coating-free, and reveal apex radii of around 15 nm when fabricated at optimized parameters (20 keV/~100 pA). Figure 7a,b show a direct comparison of a commercial MFM tip (MESP, Bruker) [5] with the here introduced Co_3_Fe α-pillars on the CoPt sample. As evident, the FEBID probe reveals more details of the laterally alternating magnetic domains and less overall noise (a). Both aspects become even more evident by a direct 3D comparison of MFM phase images, as shown in (b), for which a 81 pA α-pillar was used for a more comparable appearance. In Figure 7c, a direct comparison of MFM-maps on a hard disc drive is presented, again for a MESP tip and a FEBID-based α-pillar. As is evident, the latter gives a higher image quality with the same magnetic domain widths but with sharper edges and reduced noise. The reason for reduced lateral sensitivity via commercial tips is attributed to two effects. First, the inherently larger apex radii (>35 nm) lead to lateral convolution of adjacent magnetic regions, which naturally reduces the lateral image resolution. Second, commercial tips revealed higher MFM phase shifts, which indicate stronger overall interactions and boost the former effect. The reason for that might also be found in the fact that such products are entirely coated with the magnetic material, instead of the sole magnetic pillar. Absolute phase shifts for α-pillars are always found lower, which suggests that the tip-sample interaction is reduced but more located due to the sharper apex, hence providing higher lateral image sharpness in both examples. The last detail concerns the reduced noise for α-pillars, even though absolute phase shifts are lower than for commercial nano-probes. This is shown in Figure 7b by two 3D MFM-maps, taken from the CoPt sample but vertically adapted to obtain a similar 3D effect. The clearly smoother appearance for FEBID tips is again attributed to the more localized tip-sample interaction, which together with the improved lateral resolution demonstrates the high performance of Co_3_Fe α-pillars.

### 2.7. Wear and Stability

**Figure 8:**
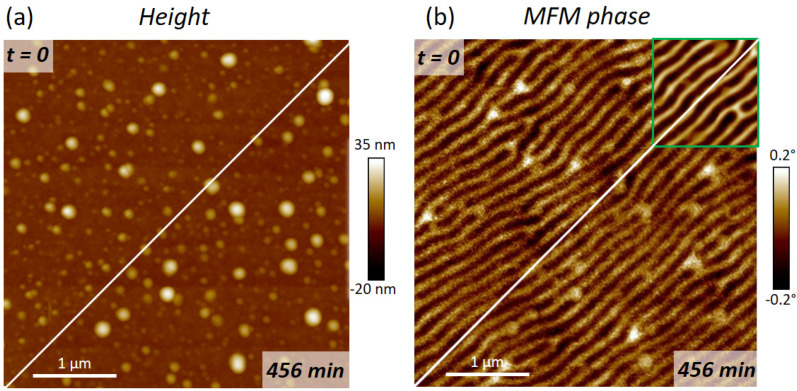
Wear testing of Co_3_Fe α-pillars, performed on a CoPt sample. The comparison shows the first and the last height image (**a**) and MFM-maps (**b**) after almost 8 h of continuous scanning along a distance of 46 cm. The green-framed box in (**b**) shows the high-quality MFM-maps, taken at 7 µm/s while the scan speed was doubled for long-time measurements, which, together with a slightly increased lift-height, explains the higher noise in (**b**).

When comparing commercial products, a closer look at wear and long-time stability is indispensably needed. Wear is a complicated quantity, as it strongly depends on sample surfaces and AFM operation conditions. To nevertheless provide quantitative information, the Co_3_Fe α-pillars were subjected to long-time measurements and long-time storage experiments. First, a slow tip velocity of 7 µm/s was chosen to acquire $4\times 4\;µm^{2}$ reference images on the CoPt sample. Next, a continuous long-time measurement was started for almost 8 h, where the scan speed was doubled (14 µm/s). Figure 8 summarizes this wear test with height images (a) and MFM-maps (b), acquired at the beginning (top left) and at the end (bottom right). The green-framed box, top left, in the MFM-map comparison (b) gives the high-quality images, taken at slow tip velocities. As evident, there is neither a loss in signal strength nor in lateral resolution or noise levels, which is truly remarkable for a total scan distance of about 46 cm. This indicates that FEBID-based Co_3_Fe α-pillars reveal low wear and high reliability due to their coating-free, all-metal character. Even if a tip gets blunt, the magnetic functionality is still present and can be laterally correlated with the height, which is one of the main problems for functionally coated standard tips.

**Figure 9:**
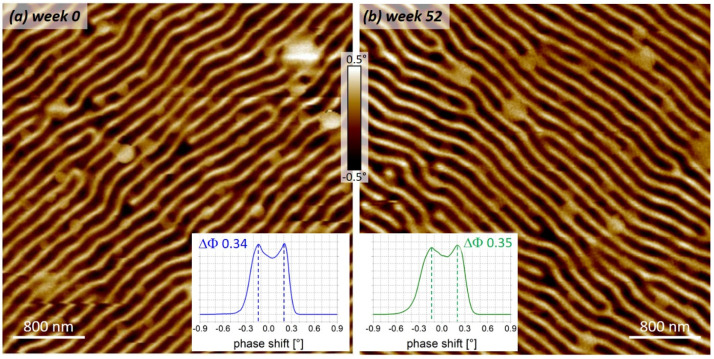
Aging tests for Co_3_Fe α-pillars. (**a**) shows an MFM-phase map of CoPt sample after fabrication, while (**b**) shows the same nano-probe on the same sample and the same AFM instrument with very similar operation parameters after a 45-weeks storage in ambient conditions. As can be seen, there is practically no loss of signal strength (compare insets, average phase shifts), image quality, or noise, which proves the chemical long-time stability of such 3D nano-probes.

As aging effects on functional properties have been observed for some FEBID materials [16,62], we stored pre-tested α-pillars for one year in ambient conditions without direct light exposure. Although its structural composition was found to be very dense (Figure 5a), oxidation could not be a priori excluded and might degrade the magnetic functionality of Co_3_Fe α-pillars. Hence, MFM experiments were repeated after exactly 12 months with the very same AFM system and identical scan parameters on the same CoPt sample. Figure 9 compares two MFM phase images, which were acquired directly after fabrication (a) and after long-time storage (b). As is evident, they are practically identical in quality and resolution, with average phase shifts of 0.34° before and after storage, as shown by the histogram insets in Figure 9. This means a practical loss-free sensitivity after one year, which underlines the chemical stability of this outstanding precursor. This concluded the evaluation of Co_3_Fe α-pillars, which reveals not only excellent MFM performance but also high wear resistance and long-time stability, as relevant for commercial applications.

## 3. Conclusions

This work introduces all-metal Co_3_Fe nano-probes for AFM-based magnetic force microscopy, fabricated by focused electron beam induced deposition. To provide mechanical rigidity, we introduce a special conical design, which is realized by gradual electron beam blurring. While the cones (called α-pillars), reveal overall heights of ~3 µm, base widths around 500 nm, and apex radii in the sub-15 nm regime for optimized performance, FEBID’s flexibility allows dimensional tuning even beyond these dimensions. Best performance was observed for primary energies of 20 keV in combination with beam currents in the range of 80–120 pA. Such nano-probes consist of densely packed nanocrystals with minimal surface contamination, although we observed the formation of an Fe-rich core along the entire pillar. Post-growth treatments by means of thermal annealing and e-beam exposure in vacuum and H_2_O environments did not improve the overall performance, which simplifies the initial fabrication routes. In comparison with well-established commercial products, optimized Co_3_Fe α-pillars reveal slightly lower absolute phase shifts, however, with very low noise and high lateral resolution for clear imaging of magnetic nanofeatures, which cannot be observed with commercial MFM probes. An expanded wear study revealed virtually no quality loss after almost 8 h of continuous operation, over which a total distance of almost half a meter was scanned. Furthermore, AFM/MFM measurements with tips stored in ambient conditions for 12 months showed that signal strength, noise level, and resolution capabilities remained the same, which underlines the high stability of FEBID-based deposits using this outstanding precursor. By that, this study lays the foundation for even more advanced, FEBID-based SPM nano-probe concepts, which might require individual, magnetic building blocks.

## 4. Methods

### 4.1. Additive Manufacturing (FEBID)

FEBID experiments and SEM analyses were conducted in an SEM/focused ion beam (FIB) dual beam microscope (Quanta 3D-FEG, Thermo Fisher, Eindhoven, The Netherlands). The precursor material (HCo_3_Fe(CO)_12_) was loaded in a standard gas injection system (GIS, Thermo Fisher, Eindhoven, The Netherlands) and heated to 65 °C at least 1 h prior to any deposition. The GIS was mounted in a high-angle port at 52° angle arranged in a vertical distance of 100 µm to the substrate and a radial distance of 150 µm relative to the deposition area. The gas flux was established at least 60 s before any deposition experiment to provide a thermodynamic equilibrium. Basic testing was done on $1\times 1\;{\mathrm{cm}}^{2}$ silicon wafers with a thermally grown, 3 nm oxide layer. For TEM studies, structures were deposited directly on TEM half grids to prevent any further preparation process. Deposition of real MFM tips was done on SS-CL platforms provided by GETec Microscopy Inc. (Vienna, AUT) [39] without any further treatments and on OTESPA-R3 cantilevers [63] (Bruker, Billercia, MA, USA), for which the original tip was truncated via FIB processing. The chamber base pressure was $\left(1.05\pm 0.2\right)\times 10^{-6}\;\mathrm{mbar}$ and stabilized at $\left(2.09\pm 0.2\right)\times 10^{-6}\;\mathrm{mbar}$ after establishing the gas flow, and substrate temperatures were kept at 21 °C. Hollow cones were 3D-printed in a circular pattern, following a previously reported protocol [24], using a stream file created by home-made C++ code. α-pillar designs were realized by a sequence of point exposures with adapted beam blurs and exposure times.

### 4.2. Morphological Characterization (SEM)

Prior to any SEM imaging, a clearance time of at least 60 min was introduced to prevent any further deposition from remaining precursor residues. Electron beam assisted purification in water vapor was performed in the Quanta 3D FEG system, using 30 keV and 2400 pA at a H_2_O partial pressure of 80 Pa in a top view arrangement with patterning strategies proposed by Seewald et al. [24]. Thermal annealing was conducted under vacuum/ambient conditions at temperatures of 250 °C and 500 °C, respectively. EBC was carried out in the same microscope at 30 keV and 2400 nA over a projected area of 0.5 µm^2^ for 5 min.

### 4.3. Structural and Chemical Characterization (TEM)

TEM was performed on (i) a Tecnai F20 (Thermo Fisher, Eindhoven, The Netherlands), operated at 200 keV in monochromated conditions, and (ii) a Titan^3^ G2 60–300 (Thermo Fisher, The Netherlands), operated at 300 keV. EDX spectra/mappings were acquired with a Super-X detector (Chemi-STEM) within the Titan system (ii). Analyses were done with the software suites VELOX (Thermo Fisher, Eindhoven, The Netherlands) and Digital Micrograph (GATAN, Pleasanton, CA, USA).

### 4.4. Atomic and Magnetic Force Microscopy (AFM/MFM)

MFM measurements were performed on a FastScanBio AFM Microscope (Bruker, Billerica, MA, USA) operated by a Nanoscope V controller. For benchmark measurements, commercially available MESP and MESP-V2 [5] (Bruker, Billerica, MA, USA) with a Co-Cr coating and a nominal tip radius of 35 nm were used. Magnetization of all MFM tips was done with a Bruker MFM “probe magnetizer”, the probes for aging experiments were re-magnetized before their usage. During MFM, lift-heights were first reduced until an attractive regime emerged, as reflected by sudden phase jumps. Afterwards, lift heights were increased again until artifact-free images were obtained. The magnetic CoPt sample was a helium beam processed multilayer system consisting of 20 blocks of Co (0.5 nm) and Pt (0.7 nm) layers [46]. The hard disk drive was an in-house device that was carefully prepared for benchmark measurements. Complementary AFM measurement for validation on a second AFM system was done on a FUSIONScope™ by Quantum Design Microscopy (Darmstadt, GER) with selected α-pillars. Analyses and post-processing of AFM data were done with the Nanoscope Suite (V1.8, Bruker, Billerica, MA, USA) and with the freeware Gwyddion (V2.62).

## Data Availability

All data, relevant for this article, are included in the figures and/or in the discussion.

## Abbreviations

AFM	atomic force microscopy
BB	beam blur
CAFM	conductive atomic force microscopy
$E_{0}$	primary electron energy
EBC	electron beam curing
ET	exposure time
FEBID	focused electron beam induced deposition
FIB	focused ion beam
FSE	forward scattered electron
GIS	gas injection system
$I_{0}$	beam current
HAADF	high-angular-annual-darkfield
MFM	magnetic force microscopy
SEM	scanning electron microscopy
SPM	scanning probe microscopy
SS-CL	self-sensing cantilever
TET	total exposure time
TEM	transmission electron microscopy
VGR	volume growth rate
EDX	energy dispersive X-ray spectroscopy
